# Fractal analysis of left ventricular trabeculations is associated with impaired myocardial deformation in healthy Chinese

**DOI:** 10.1186/s12968-017-0413-z

**Published:** 2017-12-14

**Authors:** Jiashen Cai, Jennifer Ann Bryant, Thu-Thao Le, Boyang Su, Antonio de Marvao, Declan P. O’Regan, Stuart A. Cook, Calvin Woon-Loong Chin

**Affiliations:** 1 Academic Clinical Program, Duke-NUS Medical School, Singapore, Singapore; 2 Department of Cardiology, National Heart Centre Singapore, 5 Hospital Drive, Singapore, 169609 Singapore; 3 MRC London Institute of Medical Sciences, London, UK

**Keywords:** Cardiovascular magnetic resonance, Non-compaction, Cardiomyopathy, Myocardial function

## Abstract

**Background:**

Left ventricular (LV) non-compaction (LVNC) is defined by extreme LV trabeculation, but is measured variably. Here we examined the relationship between quantitative measurement in LV trabeculation and myocardial deformation in health and disease and determined the clinical utility of semi-automated assessment of LV trabeculations.

**Methods:**

Cardiovascular magnetic resonance (CMR) was performed in 180 healthy Singaporean Chinese (age 20–69 years; males, *n* = 91), using balanced steady state free precession cine imaging at 3T. The degree of LV trabeculation was assessed by fractal dimension (FD) as a robust measure of trabeculation complexity using a semi-automated technique. FD measures were determined in healthy men and women to derive normal reference ranges. Myocardial deformation was evaluated using feature tracking. We tested the utility of this algorithm and the normal ranges in 10 individuals with confirmed LVNC (non-compacted/compacted; NC/C ratio > 2.3 and ≥1 risk factor for LVNC) and 13 individuals with suspected disease (NC/C ratio > 2.3).

**Results:**

Fractal analysis is a reproducible means of assessing LV trabeculation extent (intra-class correlation coefficient: intra-observer, 0.924, 95% CI [0.761–0.973]; inter-observer, 0.925, 95% CI [0.821–0.970]). The overall extent of LV trabeculation (global FD: 1.205 ± 0.031) was independently associated with increased indexed LV end-diastolic volume and mass (sβ = 0.35; *p* < 0.001 and sβ = 0.13; *p* < 0.01, respectively) after adjusting for age, sex and body mass index. Increased LV trabeculation was independently associated with reduced global circumferential strain (sβ = 0.17, *p* = 0.013) and global diastolic circumferential and radial strain rates (sβ = 0.25, *p* < 0.001 and sβ = −0.15, *p* = 0.049, respectively). Abnormally high FD was observed in all patients with a confirmed diagnosis of LVNC. Five out of 13 individuals with suspected LVNC had normal FD, despite NC/C > 2.3.

**Conclusion:**

This study defines the normal range of LV trabeculation in healthy Chinese that can be used to make or refute a diagnosis of LVNC using the fractal analysis tool, which we make freely available. We also show that increased myocardial trabeculation is associated with higher LV volumes, mass and reduced myocardial strain.

**Electronic supplementary material:**

The online version of this article (10.1186/s12968-017-0413-z) contains supplementary material, which is available to authorized users.

## Background

Left ventricular (LV) non-compaction (LVNC) is a clinically heterogeneous myocardial disorder characterized by increased LV trabeculation and deep inter-trabecular recesses that are in continuity with the LV cavity, but not the epicardium. In patients with LVNC, the myocardium appears as two distinct layers, consisting of a thick non-compacted endocardial layer, and a thin compacted epicardial layer [1, 2]. Though LVNC has be defined as a genetic or unclassified cardiomyopathy that is associated with heart failure and adverse cardiovascular events (including malignant arrhythmias, sudden cardiac death and thromboembolic events) [3–5], it may represent either an isolated entity or a structural trait presenting in both cardiac and non-cardiac diseases [6].

Variable degrees of LV trabeculation have been observed in other cardiac conditions, including dilated and hypertrophic cardiomyopathies [7–9], congenital heart disease [10], and in healthy individuals such as in pregnant women or athletes [6, 11, 12], suggesting that it may be a remodeling epiphenomenon or anatomical phenotype [13]. This has led to much controversy over the diagnostic criteria for LVNC, while the physiological consequences of variable degrees of LV trabeculation in healthy individuals are unknown [14, 15].

Of the modalities available for investigating LVNC, cardiovascular magnetic resonance imaging (CMR) offers a comprehensive assessment of myocardial anatomy, function, perfusion and tissue characteristics [16, 17]. Its high spatial resolution allows for better differentiation between non-compacted and compacted layers of myocardium, compared to two-dimensional (2D) echocardiography and computed tomography (CT) imaging. Though current CMR-based diagnostic criteria (Additional file 1: Table S1) are mostly based on the ratio between non-compacted and compacted myocardium/layer in terms of thickness [18, 19], mass [20] or volume [21], recent approaches and tools based on fractal geometry [22, 23] to quantify trabeculation complexity have been developed but have yet to be applied in mainstream clinical practice. Due to the wide spectrum of normal variation in trabeculation, criteria for LVNC cardiomyopathy have been developed but these are based on small sample sizes and may result in over-diagnoses [14, 24]. It is thus important to study the phenotypic variability of LV trabeculation in the normal population and develop normal reference ranges for these measures using automated and robust approaches.

Prior studies in healthy, asymptomatic population-based cohorts are equivocal on the functional consequence of LV trabeculations [25–27]. Though left ventricular ejection fraction (LVEF) is a key metric of myocardial function, its ability to detect subtle variation in myocardial function is limited. Myocardial deformation imaging offers greater insights into myocardial function with greater dimensionality [28]. Myocardial deformation is quantified using strain and strain rate, as measures of global and regional LV function, in the three cardiac planes (longitudinal, circumferential, and radial). Strain represents the change in length of the myocardium relative to its end-diastolic length (e.g. longitudinal and circumferential shortening (negative), and radial thickening (positive) during systole), while strain rate is derived as rate of such deformation [29, 30]. Integration of myocardial deformation imaging thus augments existing modalities in evaluating myocardial function and the physiological consequence of LV trabeculations in the general population.

In this study, we sought to examine the relationship between the extent of LV trabeculation, and myocardial morphology and function in healthy Chinese in Singapore using CMR to elucidate the functional and physiological consequences of LV trabeculations. We also sought to establish age- and sex-specific reference ranges for measures of LV trabeculations and myocardial strain that are currently not available in Asians. We hypothesized that the degree of LV trabeculation in healthy individuals is associated with reduced intrinsic myocardial function.

## Methods

### Study population

The study population (*n* = 180) was based on a prior study establishing comprehensive CMR reference ranges for the heart and aortic root in Singaporean Chinese [31]. To ensure adequate distribution of participants across the age range, we performed systematic recruitment of 15 to 20 individuals for each age decile in either sex. Study participants aged 20 to 69 years old, without symptoms, clinical or family history of cardiovascular or cerebrovascular disease, were prospectively recruited from the community through advertisement in the local media. Subjects were without significant comorbidities, including hypertension, hyperlipidemia or diabetes mellitus. Individuals with valvular heart disease or resting wall motion abnormalities noted on CMR were excluded from the study population.

To examine the clinical utility of reference ranges developed, confirmed and suspected LVNC cases were extracted from existing clinical CMR database at the National Heart Center Singapore (NHCS). Suspected LVNC cases are defined as patients with noncompacted to compacted (NC/C) ratio > 2.3 [18], whilst confirmed cases were those with NC/C ratio > 2.3 and at least one additional risk factor: positive family history, LV systolic dysfunction/regional wall motion abnormalities and LVNC-related complications such as arrhythmias, heart failure and thromboembolism. Exclusion criteria for all subjects included the usual contraindications to CMR: non-CMR compatible implanted cardioverter-defibrillator or pacemakers, metallic devices or foreign bodies, and severe claustrophobia.

The study was approved by the SingHealth Centralized Institutional Review Board and conducted in accordance with the Declaration of Helsinki. Written informed consent was obtained from each participant.

### Cardiovascular magnetic resonance acquisition

All participants were imaged using CMR on a 3T scanner (Ingenia, Philips Healthcare, Best, The Netherlands). Acquisition of balanced steady-state free precession cines was performed in the vertical and horizontal long axis planes, along with the sagittal LV outflow tract view (TR 2.8 to 2.9 ms; TE 1.4 to 1.5 ms; turbo factor 10; acquired voxel size 1.88 × 1.90 × 8.00 mm^3^, flip angle 45°; 40 phases per cardiac cycle). LV short axis cines extending from the atrioventricular ring to the apex were acquired subsequently to cover the entire LV and right ventricle (RV) (8 mm parallel slices with 2 mm gap; acquired voxel size 1.89 × 1.83 × 8.00 mm^3^; 30 phases per cardiac cycle).

### Image analysis

Assessment of cardiac volumes and function were performed using standardized protocols in our Research Image Analysis Laboratory (CMR42, Circle Cardiovascular Imaging Inc., Calgary, Canada) as detailed previously [31].

Myocardial strain and strain rate was measured using CMR42 (Tissue Tracking Plugin; Circle Cardiovascular Imaging Inc.). Short-axis and long-axis cine images were analyzed. LV endocardial and epicardial borders were manually delineated in the analyzed section at end-diastolic phase for subsequent tracking. The contours were then automatically propagated throughout the cardiac cycle through feature/tissue tracking by the software with strain model generation [30]. Circumferential and radial strain were measured from the LV short axis cine images, and longitudinal strain measured from vertical long axis and horizontal long axis cine images. Strain rate was obtained from myocardial strain by differentiating with respect to time. Global strain was automatically computed as the average of peak segmental strain of the entire LV, while global strain rates were similarly derived and defined separately in the both systolic and diastolic phases.

### Fractal analysis

Fractal dimension (FD), a dimensionless measure of trabeculation complexity, was measured using LV short axis cine images at end-diastole, using a semi-automated in-house fractal analysis tool in MATLAB (Mathworks Inc. Natick, Massachusetts, USA), based on an adaption of the methodology described by Captur et al. [32].

Fractal analysis was performed on each LV slice, with exclusion of the most apical slice due to partial volume effects. Each image was magnified using bicubic interpolation, and region of interest was selected by the user. Image segmentation was performed to differentiate the LV myocardium and blood pool, through a level set thresholding method as described by Li C et al. [33]. Edges of the binary image (representing endocardial border, with inclusion of trabeculations and papillary muscles) were determined using Sobel edge detection algorithm, followed by the computation of FD of the image using a box-counting method [34]. As each slice is a two-dimensional plane, the range of possible FD values for each endocardial border is between 1 and 2. This FD computation method (box-counting) was validated against fractals with known FDs (absolute mean difference: −0.01 ± 0.01; Additional file 1: Figure S1).

Global fractal characteristics were assessed through the average of the FD of each slice in the entire LV stack, and represented as global FD. For regional fractal characteristics, the LV stack is divided into apical and basal halves (with exclusion of the middle slice for odd numbered LV stacks). From the apical half of the LV, the mean apical and maximal apical FD is derived.

### Reproducibility

Inter-observer variability was determined by analysis of a randomly generated set of 20 scans by two investigators. Assessment of CMR cine images by each investigator was performed independently of the other, while intra-observer variability was assessed by repetition of the analysis after a fixed time frame (2 weeks). To evaluate the intra- and inter-observer agreement, the Intra-Class Correlation Coefficient (ICC; two-way random, agreement) was computed.

### Statistical analysis

The distribution of all continuous variables was assessed for normality using the Shapiro-Wilk test and presented either as mean ± standard deviation (SD) or median [interquartile range], as appropriate. Statistically significant clinical variables in the univariable analyses were entered in the multivariable linear regression to determine the independent association between FD and LV morphologic structures and myocardial strain. Reference ranges were defined as 95% prediction intervals using univariable linear regression between parameters and age, stratified by sex. Indeterminate regions were defined as the 95% confidence intervals of the upper and lower reference limits, to account for the effects of sample size on the reference range [35]. All statistical analyses were performed with RStudio. A two-sided *p*-value <0.05 was considered statistically significant.

## Results

CMR images of 180 healthy individuals (45 ± 13 years old; males, *n* = 91) were analyzed. Clinical characteristics and cardiac measurements (absolute and BSA-indexed values, where applicable) of the healthy subjects are shown in Table 1.

**Table 1:** Clinical and CMR Characteristics of Healthy Volunteers

	All (*n* = 180)	Male (*n* = 91)	Female (*n* = 89)	*p*
Clinical parameters				
Age, years	46.0 [34.0, 56.0]	44.0 [35.0, 55.5]	46.0 [33.0, 57.0]	0.822
Height, m	1.65 ± 0.09	1.72 ± 0.07	1.58 ± 0.06	<0.001
Weight, kg	60.8 [53.5, 75.0]	73.0 [63.2, 81.3]	54.8 [48.8, 58.7]	<0.001
Body mass index, kg/m^2^	22.7 [20.9, 25.4]	24.6 [22.3, 26.1]	21.5 [20.3, 23.1]	<0.001
Body surface area, m^2^	1.66 [1.55, 1.88]	1.87 [1.71, 1.95]	1.55 [1.47, 1.62]	<0.001
Heart rate, beats per min	75 [67, 83]	75 [67, 84]	75 [67, 82]	0.483
Systolic BP, mmHg	130 ± 16	137 ± 14	123 ± 14	<0.001
Diastolic BP, mmHg	79 ± 11	84 ± 10	74 ± 9	<0.001
Cardiovascular magnetic resonance				
LV EDV, mL	124 [108, 148]	144 [125, 161]	109 [95, 122]	<0.001
LV ESV, mL	50 [41, 61]	59 [51, 69]	41 [34, 48]	<0.001
LV SV, mL	74 [65, 87]	83 [72, 95]	66 [61, 74]	<0.001
LV mass, g	76 ± 22	93 ± 16	58 ± 10	<0.001
Mass/EDV ratio	0.59 ± 0.11	0.64 ± 0.10	0.54 ± 0.09	<0.001
LA volume, mL	85 ± 20	91 ± 19	80 ± 19	<0.001
LVEF, %	60 ± 5	58 ± 5	62 ± 5	<0.001
Indexed LV EDV, mL/m^2^	74 [67, 82]	79 [72, 86]	70 [65, 78]	<0.001
Indexed LV ESV, mL/m^2^	30 [25, 33]	32 [29, 36]	26 [24, 31]	<0.001
Indexed LV SV, mL/m^2^	44 [41, 50]	46 [41, 51]	43 [40, 49]	0.077
Indexed LV mass, g/m^2^	44 ± 9	50 ± 7	38 ± 5	<0.001
Indexed LA volume, mL/m^2^	50 ± 10	49 ± 10	51 ± 11	0.240
LV Trabeculation				
Global FD	1.205 ± 0.031	1.216 ± 0.029	1.195 ± 0.029	<0.001
Mean apical FD	1.216 ± 0.046	1.235 ± 0.040	1.197 ± 0.044	<0.001
Maximal apical FD	1.278 ± 0.045	1.293 ± 0.039	1.261 ± 0.045	<0.001
NC/C ratio (ED, LAX)	1.72 [1.60, 1.84]	1.72 [1.57, 1.86]	1.71 [1.61, 1.81]	0.749
LV Global strain				
Circumferential, %	−21.2 ± 2.8	−19.6 ± 2.1	−22.7 ± 2.6	<0.001
Radial, %	48.3 ± 10.9	42.7 ± 8.1	53.9 ± 10.5	<0.001
Longitudinal, %	−19.8 ± 2.7	−18.5 ± 2.1	−21.4 ± 2.7	<0.001
LV Global strain rate (Systolic)				
Circumferential, 1/s	−1.14 ± 0.36	−1.15 ± 0.26	−1.12 ± 0.44	0.481
Radial, 1/s	2.80 ± 1.05	2.58 ± 0.97	3.01 ± 1.09	0.001
Longitudinal, 1/s	−1.15 ± 0.25	−1.09 ± 0.21	−1.21 ± 0.27	0.001
LV Global strain rate (Diastolic)				
Circumferential, 1/s	1.42 ± 0.34	1.30 ± 0.34	1.55 ± 0.30	<0.001
Radial, 1/s	−3.43 ± 1.04	−2.99 ± 0.94	−3.88 ± 0.93	<0.001
Longitudinal, 1/s	1.28 ± 0.32	1.17 ± 0.28	1.40 ± 0.31	<0.001

*Abbreviations*: *BP* blood pressure, *LV* left ventricular, *EDV* end-diastolic volume, *ESV* end-systolic volume, *SV* stroke volume, *LA* left atrial, *FD* fractal dimension, *NC/C* non-compacted:compacted ratio, *ED* end-diastolic, *LAX* long axis view. Data presented in either mean ± standard deviation for normally distributed data or median [interquartile range] for non-normally distributed data

### Trabeculation complexity from left ventricular base to apex

**Fig. 1:**
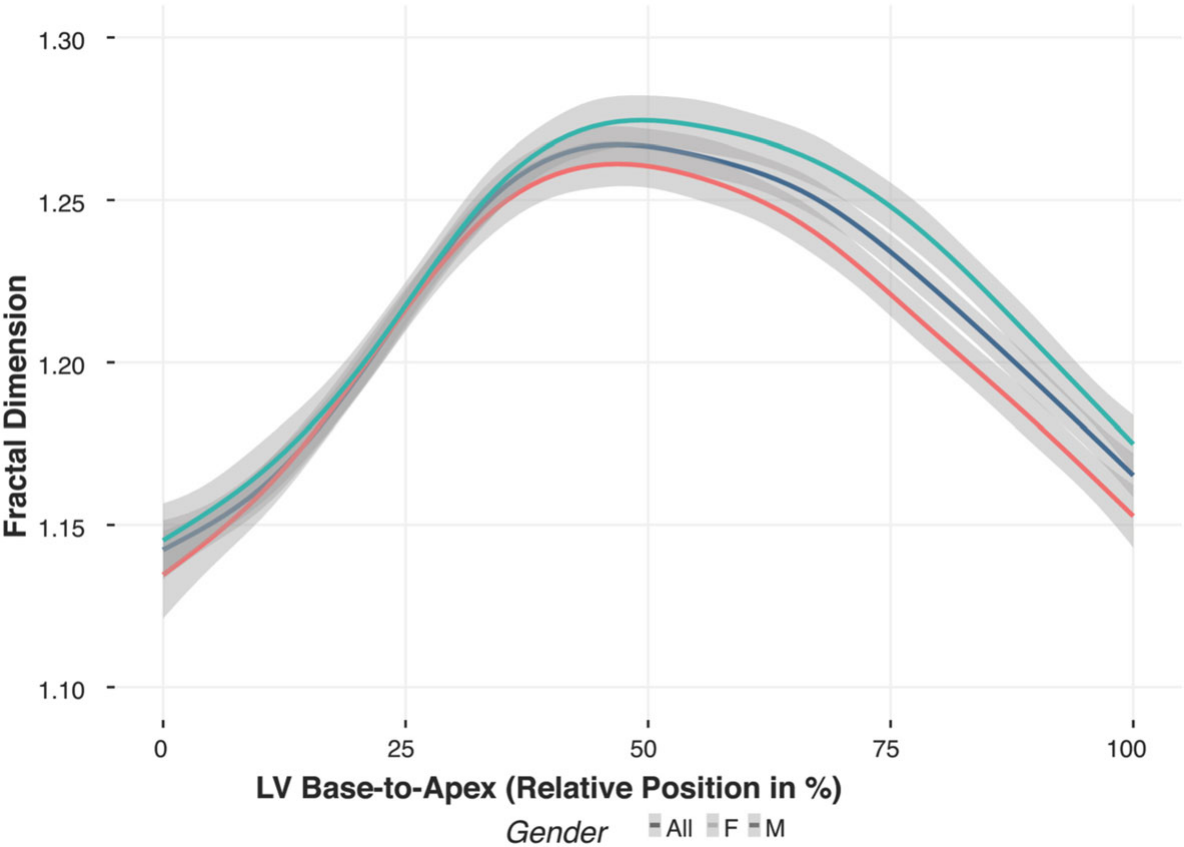
Fractal Dimension Across LV (Base-Apex) in Healthy Individuals. Pattern of left ventricular (LV) trabeculation distributed more in the apical half of the LV, where trabeculations and papillary muscles are typically located, compared to the basal half, with mid-ventricular region corresponding with the highest trabeculation complexity. Shaded region represents 95% CIs of mean values plotted

Trabeculation complexity from LV base to apex was evaluated by fractal analysis and shown in Fig. 1, revealing a pattern of LV trabeculation (higher FD) distributed more in the apical portion of the LV compared to the basal portion. The mid-ventricular region, where the papillary muscles are located, corresponded with the region of highest FD. Global FD for all healthy individuals was 1.205 ± 0.031, higher in males compared to females (1.216 ± 0.029 versus 1.195 ± 0.029, *p* < 0.0001).

### Trabeculation and demographic or anthropometric parameters

**Fig. 2:**
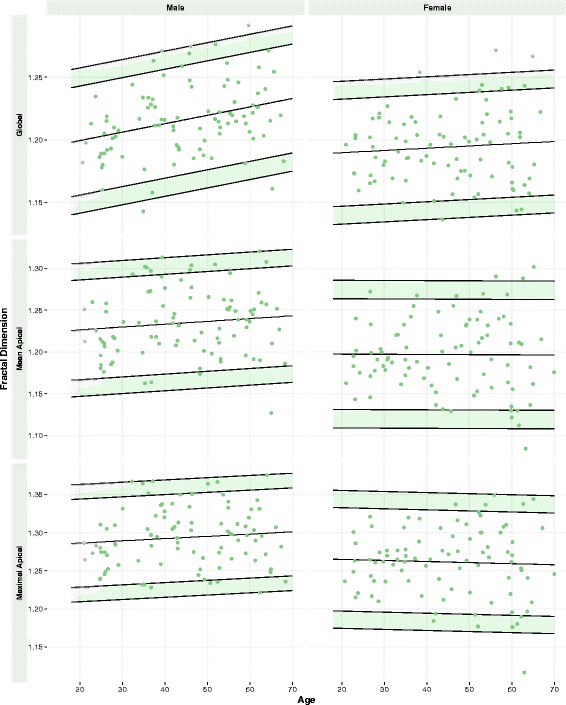
Reference Ranges for FD in Males and Females. Age- and sex-specific reference ranges for LV trabeculation measures (global and regional). Values in the shaded regions are indeterminate abnormal or borderline normal

**Fig. 3:**
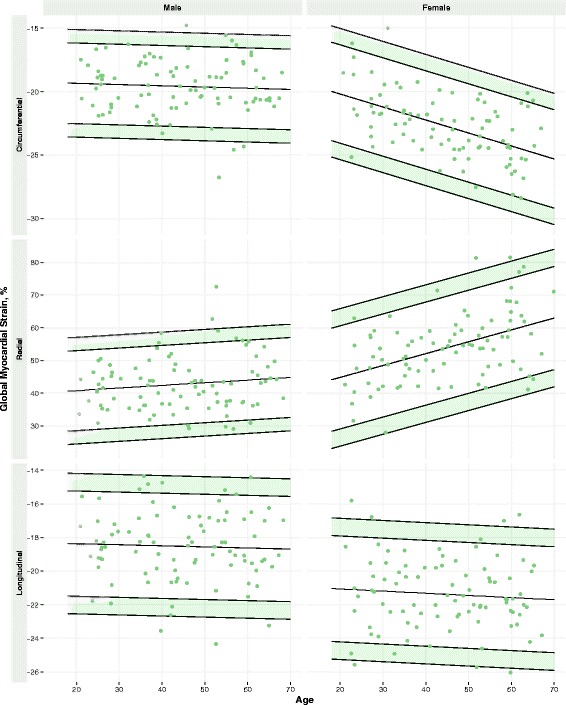
Reference Ranges for Global Myocardial Strain in Males and Females. Age- and sex-specific reference ranges for global myocardial strain in circumferential, radial and longitudinal dimensions. Values in the shaded regions are indeterminate abnormal or borderline normal

LV trabeculation extent (assessed by global FD) was positively associated with age, male sex, height, weight and body mass index (BMI) (age: standardized β, sβ = 0.18, *p* = 0.02; males: sβ = 0.35, *p* < 0.001; height: sβ = 0.20, *p* = 0.01; weight: sβ = 0.40, *p* < 0.001; BMI: sβ = 0.41, *p* < 0.001). In the multivariable regression model, age, male sex and BMI were independent determinants of global FD (*p* < 0.05 for all). Normal age- and sex-specific reference ranges for LV trabeculation and myocardial deformation measures were established (Tables 2 and 3; Figs. 2 and 3).

**Table 2:** Reference ranges in males

Males	All (*n* = 91)	20 to 29 (*n* = 19)			30 to 39 (*n* = 17)			40 to 49 (*n* = 19)			50 to 59 (*n* = 20)			60 to 69 (*n* = 16)		
	Mean ± SD	Lower	Mean	Upper	Lower	Mean	Upper	Lower	Mean	Upper	Lower	Mean	Upper	Lower	Mean	Upper
LV Trabeculation																
Global FD	1.216 ± 0.029	1.141, 1.156	1.199	1.243, 1.257	1.148, 1.163	1.206	1.250, 1.264	1.155, 1.169	1.213	1.256, 1.271	1.161, 1.176	1.220	1.263, 1.284	1.168, 1.183	1.226	1.270, 1.284
Mean Apical FD	1.235 ± 0.040	1.147, 1.167	1.226	1.286, 1.306	1.150, 1.170	1.230	1.290, 1.310	1.153, 1.173	1.233	1.293, 1.313	1.157, 1.177	1.236	1.296, 1.316	1.160, 1.180	1.240	1.300, 1.320
Max Apical FD	1.293 ± 0.039	1.209, 1.229	1.286	1.344, 1.363	1.212, 1.231	1.289	1.347, 1.366	1.215,1.234	1.292	1.350, 1.369	1.218, 1.237	1.295	1.353, 1.372	1.221, 1.240	1.298	1.356, 1.375
Global myocardial strain																
Circumferential, %	−19.6 ± 2.1	−15.1, -16.2	−19.4	−22.5, -23.6	−15.2, -16.3	−19.4	−22.6, -23.7	−15.3, -16.4	−19.5	−22.7, -23.8	−15.4, -16.5	−19.6	−22.8, -23.9	−15.5, -16.6	−19.7	−22.9, -24.0
Radial, %	42.7 ± 8.1	24.5, 28.6	40.8	53.0, 57.1	25.3, 29.4	41.6	53.8, 57.9	26.1, 30.2	42.4	54.6, 58.7	26.9, 31.0	43.2	55.4, 59.5	27.7, 31.8	44.0	56.2, 60.3
Longitudinal, %	−18.4 ± 2.3	−14.2, -15.2	−18.4	−21.5, -22.6	−14.3, -15.3	−18.4	−21.6, -22.6	−14.3, -15.4	−18.5	−21.6, -22.7	−14.4, -15.4	−18.6	−21.7, -22.7	−14.5, -15.5	−18.6	−21.8, −22.8

Upper and lower limits reflect indeterminate regions (95% CIs of reference limits)

**Table 3:** Reference ranges in females

Females	All (*n* = 89)	20–29 (*n* = 16)			30–39 (*n* = 18)			40–49 (*n* = 17)			50–59 (*n* = 20)			60–69 (*n* = 18)		
	Mean ± SD	Lower	Mean	Upper	Lower	Mean	Upper	Lower	Mean	Upper	Lower	Mean	Upper	Lower	Mean	Upper
LV Trabeculation																
Global FD	1.195 ± 0.029	1.133, 1.146	1.190	1.232, 1.247	1.134, 1.149	1.191	1.234, 1.249	1.136, 1.150	1.193	1.236, 1.250	1.138, 1.152	1.195	1.238, 1.252	1.140, 1.154	1.197	1.240, 1.254
Mean Apical FD	1.197 ± 0.044	1.109, 1.131	1.197	1.264, 1.286	1.109, 1.131	1.197	1.263, 1.286	1.108, 1.131	1.197	1.263, 1.285	1.108, 1.130	1.197	1.263, 1.285	1.108, 1.130	1.196	1.263, 1.285
Max Apical FD	1.261 ± 0.045	1.174, 1.197	1.265	1.333, 1.355	1.173, 1.196	1.263	1.331, 1.354	1.172, 1.194	1.262	1.330, 1.353	1.170, 1.193	1.261	1.329, 1.351	1.169, 1.191	1.259	1.327, 1.350
Global myocardial strain																
Circumferential, %	-22.8 ± 2.6	−15.0, -16.3	−20.2	−24.1, -25.4	−16.0, -17.3	−21.2	−25.1, -26.4	−17.1, -18.4	−22.2	−26.1, -27.4	−18.1, -19.4	−23.3	−27.1, -28.4	−19.1, -20.4	−24.3	−28.2, -29.5
Radial, %	53.9 ± 10.5	23.8, 29.1	44.8	60.6, 65.8	27.4, 32.7	48.5	64.2, 69.5	31.1, 36.3	52.1	67.8, 73.1	34.7, 39.9	55.7	71.5, 76.7	38.3, 43.6	59.3	75.1, 80.3
Longitudinal, %	−21.2 ± 2.4	−16.9, -17.9	−21.1	−24.2, -25.3	−17.0, -18.0	−21.2	−24.3, -25.4	−17.1, -18.2	−21.3	−24.5, -25.5	−17.3, -18.3	−21.5	−24.6, -25.7	−17.4, -18.4	−21.6	−24.7, -25.8

Upper and lower limits reflect indeterminate regions (95% CIs of reference limits)

### Trabeculation and left ventricular size and function

**Fig. 4:**
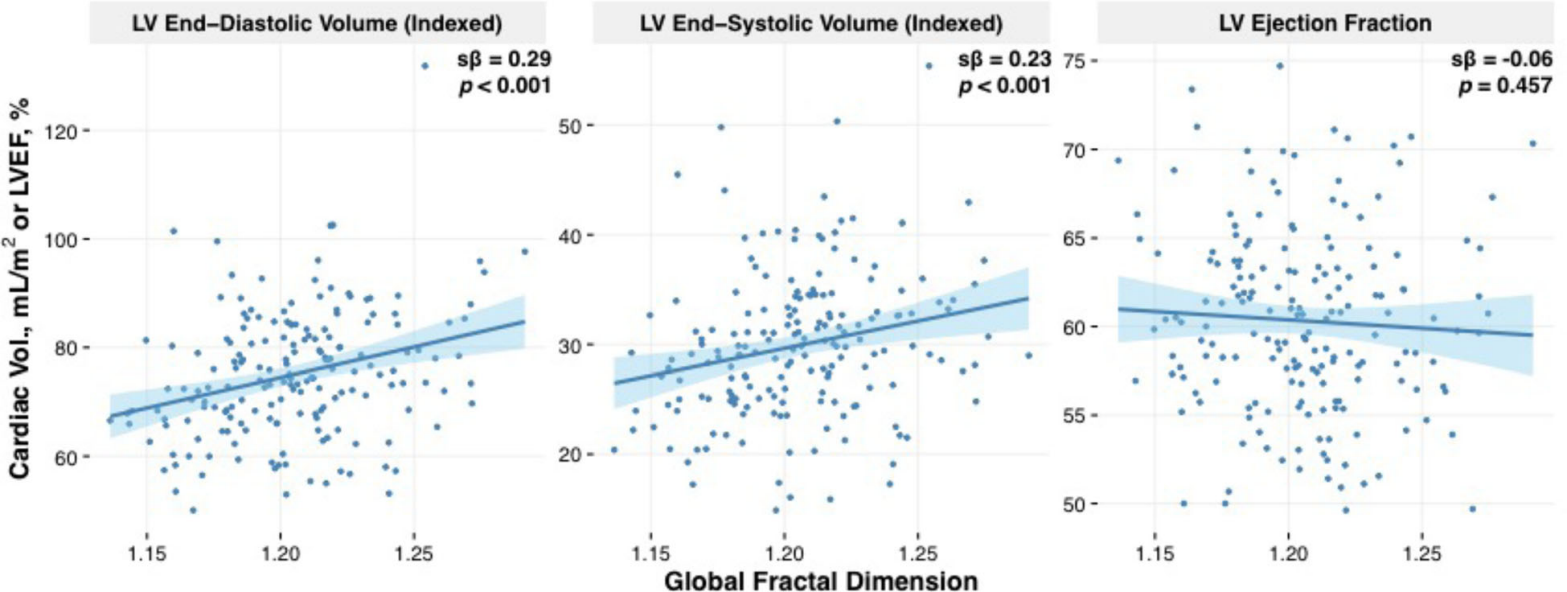
LV Trabeculation Extent (Global FD) with Cardiac Volumes and LVEF. Simple linear regression between LV trabeculation extent (global fractal dimension (FD) and cardiac volumes and LV ejection fraction in healthy population cohort. Increased indexed LV end-diastolic and end-systolic volumes with increased LV trabeculation extent. No significant association between LV ejection fraction and LV trabeculation extent. Shaded region represents 95% CIs for predicted values with linear regression

Extent of LV trabeculations was associated with indexed LV end-diastolic and end-systolic volumes (EDV, ESV), (sβ = 0.35, *p* < 0.001 and sβ = 0.23, *p* = 0.001, respectively; Fig. 4), independent of age, sex and BMI (Additional file 1: Table S2). A 10 mL/m^2^ increase in LV EDVi or ESVi was associated with a global FD increase of 0.08 and 0.20, respectively. Conversely, LV ejection fraction showed no significant association with LV trabeculation extent (sβ = −0.06, *p* = 0.46; Fig. 2). Extent of LV trabeculation was independently associated with LV mass (sβ = 0.13; *p <* 0.01), but not concentricity (mass/EDV, sβ = −0.09; *p* = 0.19) and left atrial volume (sβ = 0.12; *p* = 0.16).

### Trabeculation and myocardial deformation

Myocardial strain analysis showed that increased global FD was associated with reduced myocardial deformation across all three measures of global strain (circumferential: sβ = 0.29, *p* < 0.001; radial: sβ = −0.20, *p* = 0.008; and longitudinal: sβ = 0.24, *p* = 0.001). Global circumferential strain alone remained independently associated with global FD, after adjusting for age, sex and BMI (*p* < 0.05; Additional file 1: Table S2).

Unlike global systolic strain, global systolic strain rates showed no significant association with global FD (circumferential: sβ = 0.12, *p* = 0.121; radial: sβ = −0.04, *p* = 0.570; longitudinal: sβ = 0.05, *p* = 0.531). Conversely, global diastolic strain rates were reduced across all three dimensions (circumferential: sβ = 0.44; radial: sβ = −0.31; and longitudinal: sβ = 0.33; *p* < 0.001 for all). After adjusting for potential confounders (age, sex, body mass index), global diastolic circumferential and radial strain rates remained independently associated with global FD (*p* < 0.05 for all; Additional file 1: Table S2). Similar but weaker associations were observed between apical maximum/mean FD and impaired myocardial strains. Conversely, there were no associations between NC/C ratio and LV volumes, myocardial mass and all measures of myocardial deformation (*p* > 0.05 for all).

### Clinical utility of reference ranges

**Fig. 5:**
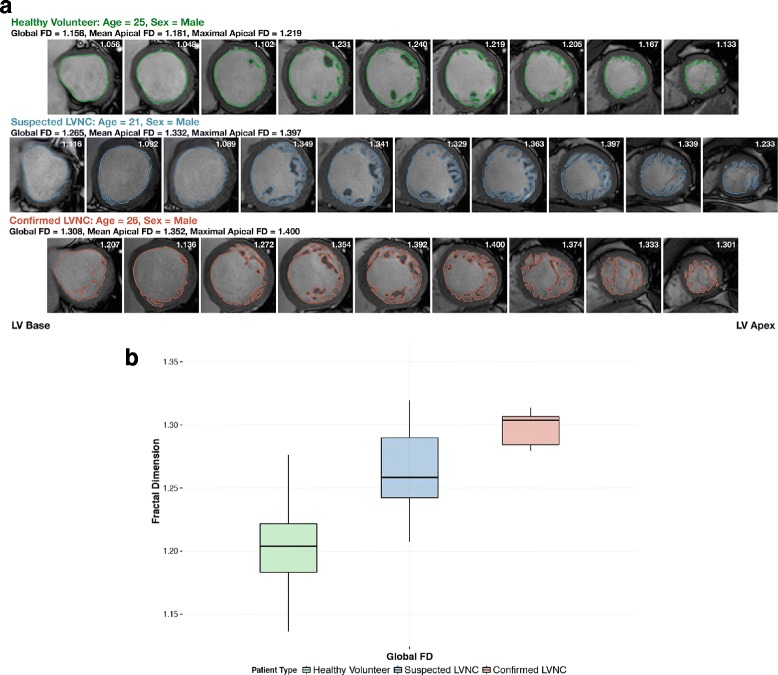
Fractal Analysis in Healthy Subjects, Confirmed LVNC and Suspected LVNC. **a** LV trabeculation extent measured using fractal analysis of LV short axis images in three representative subjects: healthy subject (first row), confirmed LVNC (second row), suspected LVNC with increased LV trabeculation (third row). Global FD value of each individual slice was presented at the top right corner. **b** Confirmed LVNC patients had significantly higher global FD values compared to individuals with suspected LVNC and healthy subjects (*P* < 0.0001 for comparison). Data presented in box and whisker plots (Tukey method)

All confirmed LVNC patients (*n* = 10; age: 33 ± 15 years; males = 10) had higher than normal FD values based on age- and sex-specific FD reference ranges we had established (global FD: 1.288 ± 0.033). The global FD was 1.270 ± 0.045 in those with suspected LVNC (*n* = 13; age: 24 ± 12 years; males = 12). Of note, five patients with suspected LVNC had normal FD (38%), despite a NC/C ratio of >2.3 (Fig. 5).

### Intra- and inter-observer variability

Reproducibility of FD and myocardial deformation measures was assessed in 20 patients and presented in Table 4. Fractal analysis showed good reproducibility with high ICC for global FD (intra-observer: 0.924, 95% confidence interval (CI) 0.761–0.973; inter-observer: 0.925, 95% CI 0.821–0.970), compared to long axis end-diastolic NC/C ratio, which had moderate ICCs (intra-observer: 0.921, 95% CI 0.815–0.968; inter-observer: 0.499, 95% CI 0.085–0.766). Strain had high intra- and inter-observer agreement throughout all three measures (circumferential, radial and longitudinal), while strain rates were generally had high reproducibility in circumferential and radial axes, and moderate reproducibility in the longitudinal axis.

**Table 4:** Intra- and inter-observer variability of LV trabeculation and myocardial deformation measures

	Intra-observer ICC		Inter-observer ICC	
	ICC	95% CI	ICC	95% CI
Fractal dimension				
Global FD	0.924	0.761–0.973	0.925	0.821–0.970
Mean Apical FD	0.901	0.729–0.962	0.930	0.832–0.972
Max Apical FD	0.946	0.864–0.978	0.969	0.899–0.989
NC/C Ratio				
End-Diastolic NC/C (LAX)	0.921	0.815–0.968	0.499	0.085–0.766
Strain				
Circumferential (LVSA)	0.924	0.820–0.969	0.825	0.525–0.933
Radial (LVSA)	0.965	0.916–0.986	0.904	0.716–0.964
Longitudinal (VLA, HLA)	0.709	0.393–0.876	0.765	0.488–0.901
Strain rate (Systolic)				
Circumferential (LVSA)	0.926	0.822–0.970	0.900	0.763–0.959
Radial (LVSA)	0.919	0.809–0.967	0.924	0.818–0.969
Longitudinal (VLA, HLA)	0.467	0.057–0.746	0.463	0.028–0.748
Strain rate (Diastolic)				
Circumferential (LVSA)	0.862	0.688–0.943	0.723	0.290–0.893
Radial (LVSA)	0.849	0.662–0.937	0.853	0.665–0.939
Longitudinal (VLA, HLA)	0.474	0.057–0.752	0.561	0.160–0.801

*HLA* horizontal long axis, *SA* short axis, *VLA* vertical long axis

## Discussion

This study examined LV trabeculation in healthy Singaporean Chinese to define normal age- and sex-specific ranges and determined the association of FD extent with cardiac physiology. The extent of LV trabeculation is higher in males, increased with age and BMI. Increased trabeculation was associated with increased LV volumes, myocardial mass and impaired myocardial strain (global circumferential, diastolic circumferential and radial strain rate), independent of age, sex and BMI.

The clinically heterogeneous nature of LVNC has led to much difficulty in achieving a consensus over the diagnostic criteria, which are currently predominantly based on semi-quantitative LV morphology assessment. A recent classification grouped LVNC into 7 different entities, of which the presence of non-compacted (trabeculated) morphology with normal systolic and diastolic function, size and wall thickness is termed as isolated LVNC [6]. In an otherwise healthy population, the extent of hypertrabeculation was not associated with deterioration in cardiac function over more than 9 years of follow-up [26]. Of note, the study assessed LV trabeculation with the conventional NC/C ratio that may be less sensitive and has suboptimal reproducibility. More studies are needed to confirm the prognostic implications of increased LV trabeculations, preferably using more accurate and precise approaches.

Fractal analysis is a novel and more objective approach of assessing the extent of LV trabeculations. Captur et al. had developed a fractal analysis plugin for the OsiriX program [23]. Our semi-automatic fractal analysis algorithm is based on similar principles of box-counting and segmentation, but less complex and more automatic. Only the selection of ROI was manually defined by the user. This approach resulted in excellent intra−/inter-observer reproducibility and accuracy when validated against fractals of known FD. The fractal analysis tool is now freely available as a standalone package on GitHub (10.5281/zenodo.836797),and not limited as a plugin developed specifically for any analysis program.

In a recent study, Captur et al. demonstrated no association between FD and age, sex and allometric parameters. The extent of LV trabeculations varied with ethnicities, highest FD in African Americans and Hispanics and least in Chinese Americans [27]. These observations were made in participants with BMI <25 kg/m^2^ and older participants >45 years. Our population was more homogeneous (Singaporean Chinese) with a much wider age range (20–69 years). At least 15–20 individuals were systematically recruited in each age decile in either sex. We reported higher FD values in males compared to females and an association between FD and age, particularly in males. It is likely these findings were not observed in the MESA population because of the narrower age range in their study. The maximal apical FD in our study was 1.278 compared to 1.197 in Chinese Americans [27]. These differences can be explained by the different methodologies in fractal assessment. The field strength used in the two studies are different that theoretically, may affect the spatial resolution of LV trabeculations and FD values. These observations highlighted the importance of establishing normal FD reference ranges specific to the population, fractal analysis tool and CMR platform. We tested the reference ranges in a group of patients with confirmed and suspected LVNC. All confirmed LVNC patients (NC/C ratio > 2.3 and at least 1 risk factor) had abnormally high FD values. Conversely, 38% of patients with suspected LVNC had normal FD that suggest they may have been misclassified as LVNC based on NC/C ratio.

Using fractal analysis as a more sensitive technique of assessing LV trabeculation, we examined the association between extent of LV trabeculation and myocardial structure and function in the healthy subjects. Unlike NC/C ratio, FD as a measure of LV trabeculation was associated with increased cardiac volumes and LV mass (more eccentric hypertrophy phenotype), but not LV concentricity. Although reduction in myocardial deformation has been shown in LVNC patients [36–39], our study demonstrated progressive impairment in regional circumferential strain with LV trabeculation extent even in healthy individuals. This was consistent with recent findings by Kawel et al. [39], and we further demonstrated an impairment in diastolic relaxation with increased LV trabeculation in healthy subjects. Of note, NC/C ratio lacked any associations with strain parameters, underscoring the limitations of NC/C ratio. In normal hearts, trabeculae provide an active mechanical leverage during systole [40]. It remains unclear if hypertrabeculation leads to deterioration of myocardial function or an epiphenomenon of adaptation to cardiac loading and other hemodynamic conditions. LV volumes increased in response to myocardial stress, causing trabecular muscles to become more prominent and increased trabeculations. This process may be reversible in some but in others, the heart may decompensate and fail [41]. Based on insights from myocardial fiber orientation, the increased trabeculations likely involve remodeling of the mid-myocardial layer, where circumferential fibers are located [28, 42]. Reduced diastolic strain rates with increased LV trabeculations suggest an association with abnormal relaxation and non-compliance of the LV, as evident in LVNC patients [43, 44].

### Study limitations

As fractal dimension provides a dimensionless representation of endocardial trabecular complexity, it may account for only part of the LVNC phenotype – prominent LV trabeculation and deep inter-trabecular recesses [22]. The role of a thin compacted epicardial layer in the LVNC phenotype could be of diagnostic importance and has not been accounted for in fractal analysis [6, 45]. The number of patients referred with possible LVNC in the study was relatively small because it is not a very common cardiac condition. Therefore, we were not able to establish the best FD measure (global, mean or maximum apical FD) that discriminates between normal and LVNC. Although of the three measures, global FD demonstrated the strongest and independent association with impaired myocardial deformation.

## Conclusions

We define here the normal FD reference ranges in healthy Chinese, which is of clinical utility for diagnosing LVNC and understanding normal variation of LV trabeculations in health. We show that increased myocardial trabeculation is independently associated with increased LV volumes, myocardial mass and reduced myocardial strain that could indicate the presence of subclinical myocardial dysfunction. This suggests increased LV trabeculations likely represents a continuum of functional effects in health and disease.

## Additional file

Additional file 1: Online supplemental data. (DOCX 379 kb)

## Abbreviations

2D: 2-dimensional
CI: Confidence interval
CMR: Cardiovascular magnetic resonance
Ecc: Regional systolic circumferential strain
EDVi: End-diastolic volume index
ESVi: End-systolic volume index
FD: Fractal dimension
ICC: Intra-class correlation coefficient
IQR: Interquartile range
LV: Left ventricle/ventricular
LVNC: Left ventricular non-compaction
MESA: Multi-Ethnic Study of Atherosclerosis
NC/C: Non-compacted to compacted ratio
RV: Right ventricle/ventricular
SD: Standard deviation
TE: Echo time
TR: Repetition time
