# Supplementary material for: Fractal analysis of left ventricular trabeculations is associated with impaired myocardial deformation in healthy Chinese
Source: J Cardiovasc Magn Reson. 2017 Dec 14;19:102. doi: 10.1186/s12968-017-0413-z (PMC5729602; doi:10.1186/s12968-017-0413-z)
Supplement: Additional file 1: — Online supplemental data. (DOCX 379 kb) [file 12968_2017_413_MOESM1_ESM.docx]

**SUPPLEMENTAL MATERIAL**

**Fractal Analysis of Left Ventricular Trabeculations is Associated with Impaired Myocardial Deformation in Healthy Chinese**

Supplemental Figure 1: Comparison of Theoretical and Measured Fractal Dimensions (FD) of Known Fractals


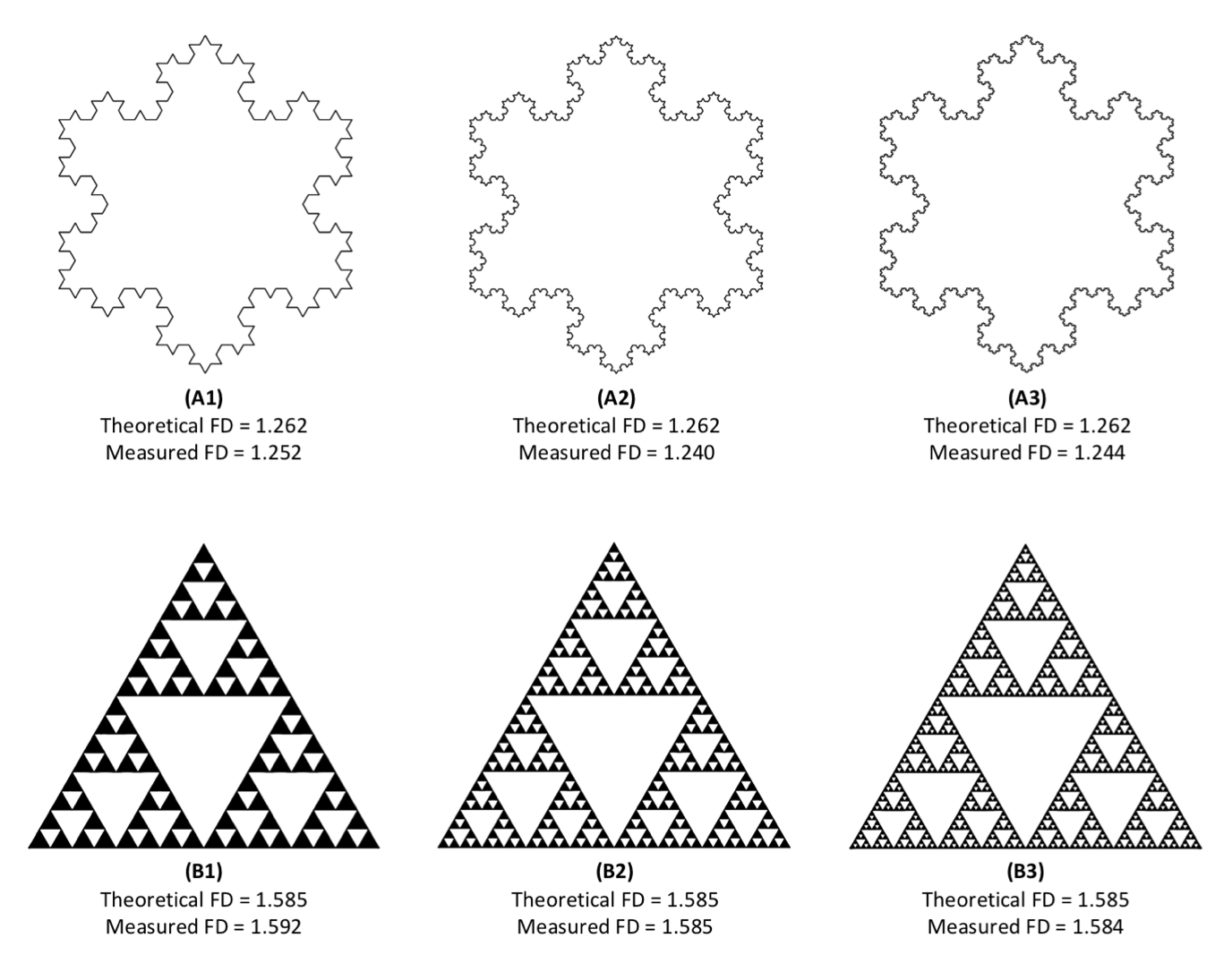


(A) Koch snowflake (A1: 4th iteration, A2: 5th iteration, A3: 6th iteration); (B) Sierpinski triangle (B1: 4th iteration, B2: 5th iteration, B3: 6th iteration)

Supplemental Table 1: Existing CMR-based Criteria for LVNC

| Criteria by | Parameters | Description |
| --- | --- | --- |
| Petersen et al.(1) | NC/C ratio  at end-diastole | Analysis of three long-axis views (4CH, VLA, LVOT), with non-compaction/hypertrabeculation identified as a distinct two-layered structure of trabeculated and compacted myocardium. Ratio of non-compacted to compacted myocardium (NC/C ratio) in end-diastole measured from segments with the most pronounced trabeculations in each of the three long-axis views, with the maximal ratio used for analysis. |
| Stacey et al.(2) | NC/C ratio at end-systole | Analysis of short-axis cine views, with the exclusion of papillary muscles or its attachments as trabeculations. NC/C ratio measured from the region with the largest proportion of trabeculated to compacted myocardium in end-systole. |
| Jacquier et al.(3) | LV trabecular mass, total LV mass at end-diastole | Analysis for short-axis cine views, with measurement of trabeculated LV mass and total LV mass, through outlining of epicardial and endocardial contours, with the inclusion of papillary muscles in the myocardial mass. |
| Captur et al.(4) | Global FD  at end-diastole | Fractal analysis performed on end-diastolic frames of each short-axis stack, with global LV trabecular complexity measured as a continuous variable, fractal dimension (FD). Image segmentation performed with automated thresholding technique and subsequent calculation of FD using the box-counting method. Global LV FD is assessed through averaging the FD of each slice in the LV. |

Supplemental Table 2: Multivariable Regression Models

|  | Global FD | Age | Gender (Male) | BMI |
| --- | --- | --- | --- | --- |
| Cardiac Volumes | | | | |
| LVEDVi | 133.62 | -0.33 | 5.94 | -0.63 |
|  | p<0.001 | p<0.001 | p<0.001 | p=0.009 |
| LVESVi | 49.11 | -0.17 | 5.73 | -0.41 |
|  | p=0.001 | p<0.001 | p<0.001 | p=0.002 |
| LVEF | 4.61 | 0.05 | -4.66 | 0.19 |
|  | p=0.727 | p=0.069 | p<0.001 | p=0.096 |
| Global Strain | | | | |
| Circumferential | 15.44 | -0.06 | 2.81 | -0.01 |
|  | p=0.013 | p<0.001 | p<0.001 | p=0.915 |
| Radial | -33.15 | 0.23 | -10.64 | 0.131 |
|  | p=0.185 | p<0.001 | p<0.001 | p=0.545 |
| Longitudinal | 5.97 | -0.01 | 2.79 | -0.03 |
|  | p=0.310 | p=0.345 | p<0.001 | p=0.601 |
| Global Strain Rate (Systolic) | | | | |
| Circumferential | 1.02 | 0.0007 | 0.02 | -0.01 |
|  | p=0.132 | p=0.631 | p=0.657 | p=0.336 |
| Radial | -0.82 | 0.01 | -0.50 | 0.03 |
|  | p=0.747 | p=0.046 | p<0.001 | p=0.112 |
| Longitudinal | 0.25 | -0.002 | 0.15 | -0.01 |
|  | p=0.708 | p=209 | p<0.001 | p=0.039 |
| Global Strain Rate (Diastolic) | | | | |
| Circumferential | -2.77 | -0.01 | -0.17 | -0.01 |
|  | p<0.001 | p<0.001 | p<0.001 | p=0.211 |
| Radial | 5.12 | 0.01 | 0.78 | 0.002 |
|  | 0.049 | 0.155 | p<0.001 | 0.898 |
| Longitudinal | -1.26 | -0.01 | -0.19 | -0.01 |
|  | p=0.097 | p<0.001 | p<0.001 | p=0.146 |

# References

1. Petersen SE, Selvanayagam JB, Wiesmann F et al. Left ventricular non-compaction: insights from cardiovascular magnetic resonance imaging. J Am Coll Cardiol 2005;46:101-5.

2. Stacey RB, Andersen MM, St Clair M, Hundley WG, Thohan V. Comparison of systolic and diastolic criteria for isolated LV noncompaction in CMR. J Am Coll Cardiol Img 2013;6:931-940.

3. Jacquier A, Thuny F, Jop B et al. Measurement of trabeculated left ventricular mass using cardiac magnetic resonance imaging in the diagnosis of left ventricular non-compaction. Eur Heart J 2010;31:1098-104.

4. Captur G, Muthurangu V, Cook C et al. Quantification of left ventricular trabeculae using fractal analysis. J Cardiovasc Magn Reson 2013;15:36.
